# Triphala and Its Active Constituent Chebulinic Acid Are Natural Inhibitors of Vascular Endothelial Growth Factor-A Mediated Angiogenesis

**DOI:** 10.1371/journal.pone.0043934

**Published:** 2012-08-24

**Authors:** Kai Lu, Debanjan Chakroborty, Chandrani Sarkar, Tingting Lu, Zhiliang Xie, Zhongfa Liu, Sujit Basu

**Affiliations:** 1 Department of Pathology, Ohio State University, Columbus, Ohio, United States of America; 2 Division of Pharmaceutics, College of Pharmacy, Ohio State University, Columbus, Ohio, United States of America; 3 Arthur G. James Comprehensive Cancer Center, Ohio State University, Columbus, Ohio, United States of America; 4 Dorthy M. Davis Heart and Lung Research Institute, Ohio State University, Columbus, Ohio, United States of America; Children's Hospital Boston & Harvard Medical School, United States of America

## Abstract

Triphala churna (THL) is a combination of three fruits that has been used for many years in India for the treatment of various diseases. There are now reports which indicate that THL can inhibit growth of malignant tumors in animals. However, the mechanisms by which THL mediates its anti-tumor actions are still being explored. Because vascular endothelial growth factor-A (VEGF) induced angiogenesis plays a critical role in the pathogenesis of cancer, we therefore investigated whether tumor inhibitory effects of THL or its active constituents are through suppression of VEGF actions. We herein report that THL and chebulinic (CI) present in THL can significantly and specifically inhibit VEGF induced angiogenesis by suppressing VEGF receptor-2 (VEGFR-2) phosphorylation. These results are of clinical significance as these inexpensive and non-toxic natural products can be used for the prevention and treatment of diseases where VEGF induced angiogenesis has an important role.

## Introduction

Angiogenesis or new blood vessel formation is required for the initiation, growth and progression of malignant tumors [1]–[4]. This is a tightly regulated process and a shift in the balance towards pro-angiogenic molecules results in activation of the angiogenic switch [1]–[4]. Among the pro-angiogenic molecules, VEGF is essential for tumor angiogenesis [1]–[4]. VEGF mediates its effects mainly through VEGFR-2, which in turn stimulates proliferation, migration of endothelial cells and leakiness of neovessels [1]–[4].

Triphala churna is a powdered preparation of three myrobalan fruits, *Emblica officinalis* Gaertn (*Amla*), *Terminalia chebula* Retz (*Haritaki*) and *Terminalia belerica* Roxb (*Bibhitaki*) in equal proportions [5]–[7]. This fruit formulation has been extensively used in the traditional Indian system of medicine, Ayurveda for the treatment of several disorders of the gastrointestinal and cardiovascular systems [5]–[8]. In addition, THL is also consumed by the people of Indian subcontinent for its high nutritional value [8]. Recently, it has been demonstrated that THL can inhibit the growth of carcinogen induced stomach cancer, murine thymic lymphoma and human pancreatic cancer in mice [7]–[10]. However, there is no mention in these reports regarding the effects of THL on tumor angiogenesis [7]–[10].

High performance liquid chromatography (HPLC) has revealed gallic acid (GA), ellagic acid (EA) and chebulinic acid (CI) to be the major constituents of THL [6], [8]. The plasma levels of GA and EA after oral intake of fruits containing these two compounds, and thereby their bioavailability has been reported to be poor [11], [12]. As THL has been shown to inhibit malignant tumor growth in animals [7]–[10], therefore there is a possibility that other bioactive compounds present in THL mediate its anti-tumor effects in these animals. Because VEGF induced angiogenesis is required for the growth of malignant tumors [1]–[4], we thus investigated if THL and or CI have any effects on VEGF mediated angiogenesis.

## Materials and Methods

### Reagents

**Figure 1:**
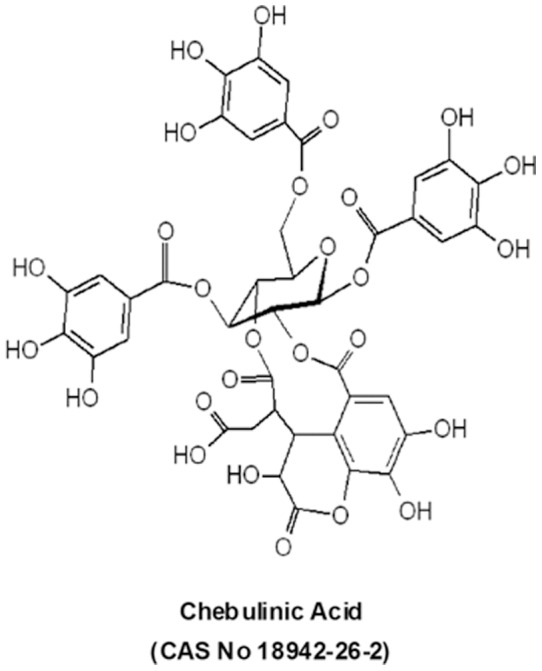
The chemical structure of chebulinic acid.

THL was from Dabur India, New Delhi, India and >90% pure CI was from Natural Remedies, Bangalore, India (Fig. 1). Recombinant human VEGF-A was from R&D systems, MN, USA. THL and CI solutions used were endotoxin free as tested by gel-clot limulus amebocyte lysate method with reagents from Charles River, MA, USA [13].

**Figure 2:**
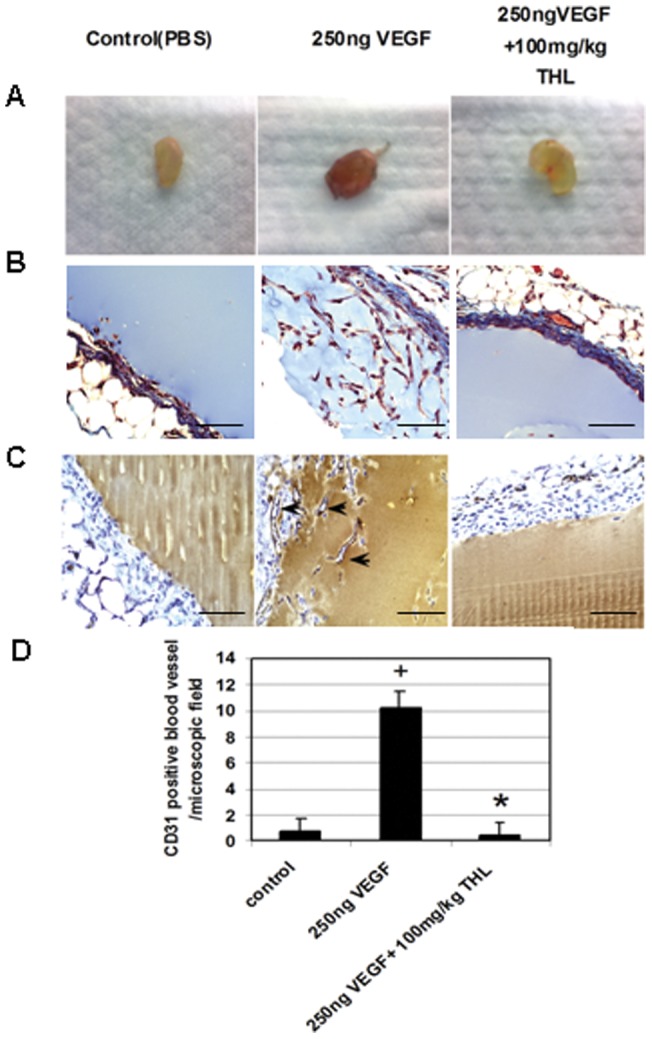
The effects of THL (triphala churna) on *in vivo* matrigel angiogenesis assay. (**A**) Photographs of representative matrigel plugs show THL untreated red colored vascular endothelial growth factor (VEGF) containing plugs in comparison to the VEGF minus PBS containing controls. (**A**) In contrast, THL (triphala churna) treated VEGF containing matrigel plugs were pale. (**B**) Masson's trichrome staining (endothelial cells stain red and matrigel stains blue) and (**C, D**) CD31 immunohistochemistry of the matrigel plug sections show large numbers of endothelial cells in THL untreated VEGF containing plugs in comparison to controls (+, *p*<0.05). In contrast, THL treated VEGF containing matrigel plug section has considerably low numbers of endothelial cells as detected by Masson's trichrome staining and CD31 staining (*, p<0.05). Scale bars in **B** and **C**, 50 μm. *n* =  six for each experimental group.

### Matrigel plug assay

All animal experiments were performed after approval by the Institutional Animal Care and Use Committee. Matrigel (cat# 356231, BD Biosciences, CA, USA) was subcutaneously (s.c.) injected either alone or mixed with VEGF (250 ng) in a total volume of 600 ml into the ventral flanks of 6–8 wks old male C57BL6 mice. Thereafter, these animals were treated with a single daily dose of 100 mg/kg of THL by gavage for 7 days. On day 8, the matrigel plugs were removed and the matrigel sections were either stained with Masson's trichrome or immunohistochemistry was performed using CD31 rabbit polyclonal antibody (Abcam, MN, USA) [14]–[17].

**Figure 3:**
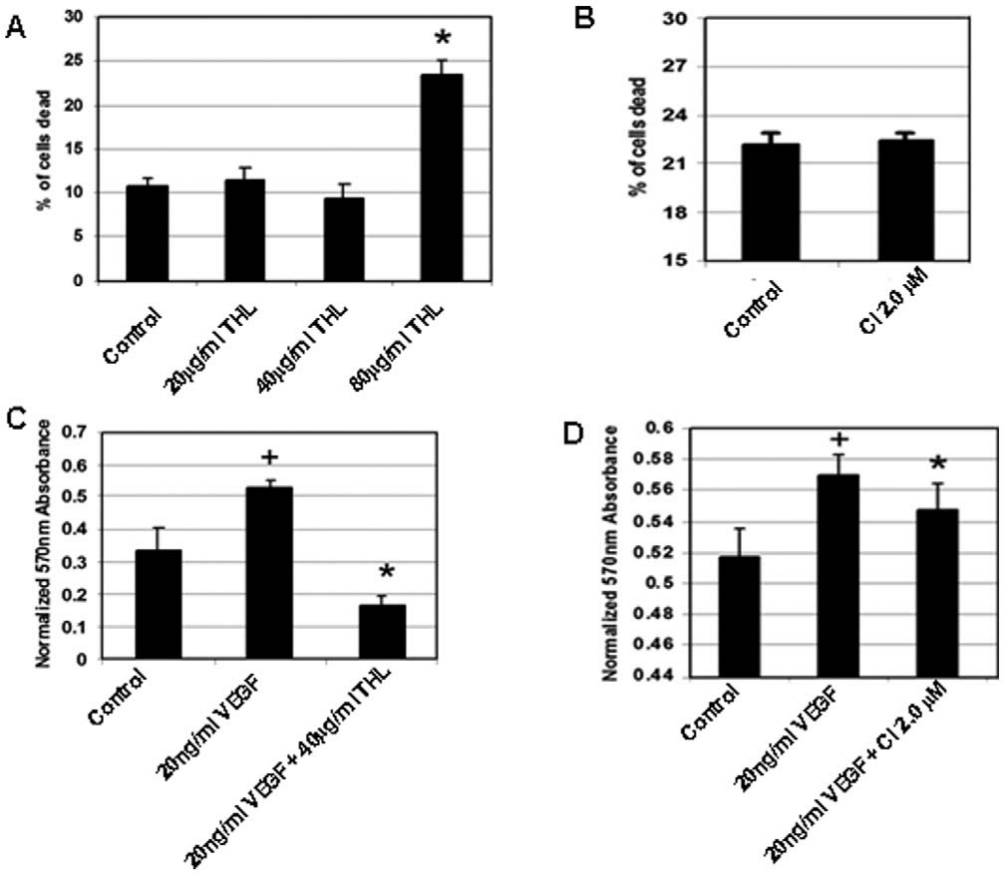
The effects of THL (triphala churna) and chebulinic acid (CI) on endothelial cell viability. (**A**) The cytotoxic efffects of various concentrations of THL (triphala churna) and (**B**) chebulinic acid (CI) on human umbilical vein cells (HUVEC) (*, *p<*0.05). (**C**) Effects of THL and (**D**) CI on HUVEC proliferation stimulated by vascular endothelial growth factor (VEGF) (+, *p<*0.05 versus vascular endothelial growth factor (VEGF) and untreated control). The stimulatory effect of VEGF on HUVEC was abrogated by THL and CI (*, *p<*0.05). All error bars represent SEM. Results shown are representative of six separate experiments.

### Quantification of CI in mouse plasma and THL

Blood was collected from mice following gavaging them with a single dose of THL (100 mg/kg). Thereafter, an aliquot of 10 μL mouse plasma collected at different time intervals from these animals were spiked into 100 μL normal mouse plasma containing 1000 ng/mL hesperetin (Sigma, MO, USA) as internal standards. The mixture was vortexed for 30 sec and centrifuged at 14,000 *g*. The CI was then extracted by ethyl acetate followed with evaporation to dryness. The acetonitrile (Sigma, MO, USA) dissolved in the intermediated stock solutions of calibration curve samples was prepared in parallel with the mouse plasma samples. The residue was reconstituted in mobile phase and thereafter analyzed by LC-MS/MS system [6].

**Figure 4:**
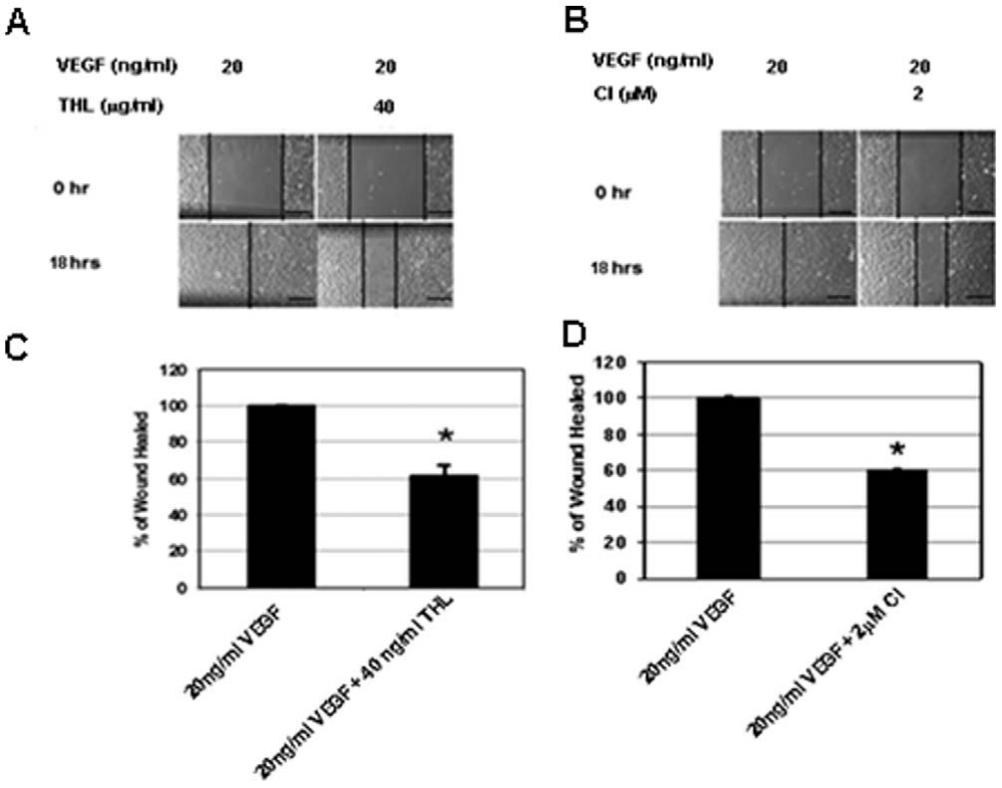
The effects of THL (triphala churna) and chebulinic acid (CI) on vascular endothelial growth factor (VEGF) induced migration of human umbilical vein cells (HUVEC). Phase-contrast microphotographs of the wound area in HUVEC monolayer at 18 h after wounding (**A–D**) VEGF promotes complete wound closure or healing in 18vh. In contrast, this effect is lost when cells are treated either with THL (**A, C**) or CI (**B, D**). Wound healing is calculated as the distance covered by cells in relation to the initial wound distance at 0 h and is expressed as a percentage of the initial distance at 0 h. *, *P*<0.05. All error bars represent SEM. Scale bars in **A** and **B**, 200 μm. Results shown are representative of six separate experiments.

**Figure 5:**
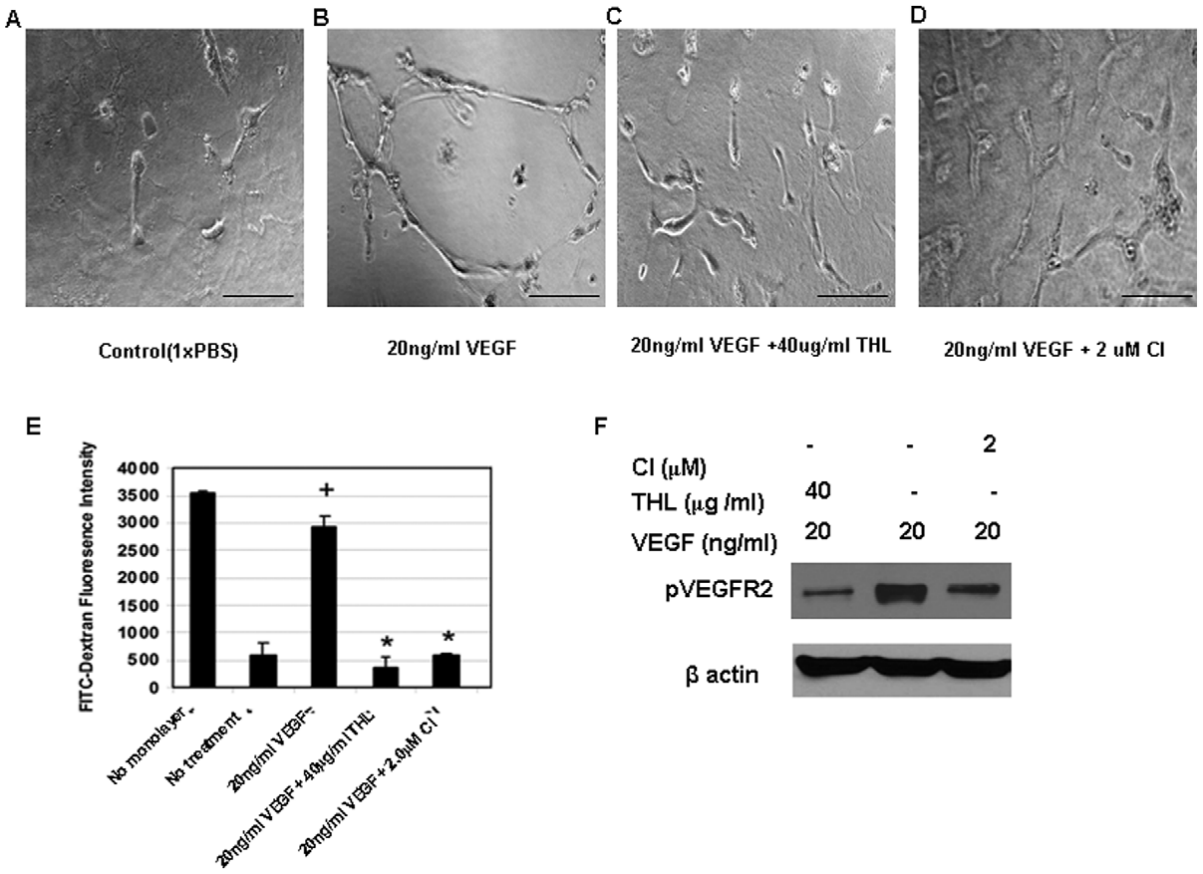
The effects of Triphala churna (THL) and chebulinic acid (CI) on vascular endothelial growth factor (VEGF) induced tube formation and permeability in human umbilical vein cells (HUVEC). In contrast to (**A**) control, (**B**) VEGF promotes tube formation in HUVEC. However, treatment with either (**C**) THL or (**D**) CI inhibits VEGF induced tube formation in HUVEC. (**E**) Similarly, VEGF induces significant permeability in HUVEC in comparison to untreated control (+, *p*<0.05). On the contrary, THL and CI significantly inhibit VEGF mediated permeability in HUVEC (*, *p*<0.05). (**F**) Western blot analysis shows significant inhibition of vascular endothelial growth factor receptor-2 (VEGFR2) phosphorylation on treatment with THL and CI. Scale bars in **A–D**, 200 μm. The figure is representative of six separate experiments.

LC-MS/MS Condition: Biosystems Sciex API 3000 mass spectrometer (Applied Biosystems Sciex, Ontario, Canada) equipped with an electrospray ionization (ESI) source and SIL-10ADvp Shimadzu HPLC system (Shimadzu, Columbia, MD, USA) were applied for mass analysis and sample analysis. The detector was operated in MRM mode using the transitions from the protonated molecular ions to product ions at m/z 955.00/337.3; 953/301.1 for CICA, respectively, and the m/z 303.10/153.10 for I.S. The chromatographic separation was then performed using a Beta Basic C8 column (2.1 mm×50 mm, 5 µm, Thermo Hypersil-Keystone, Bellefonte, PA) and the mobile phases of pump B with acetonitril, and pump A with H2O [0.2% formic acid (Sigma, MO, USA)].

**Figure 6:**
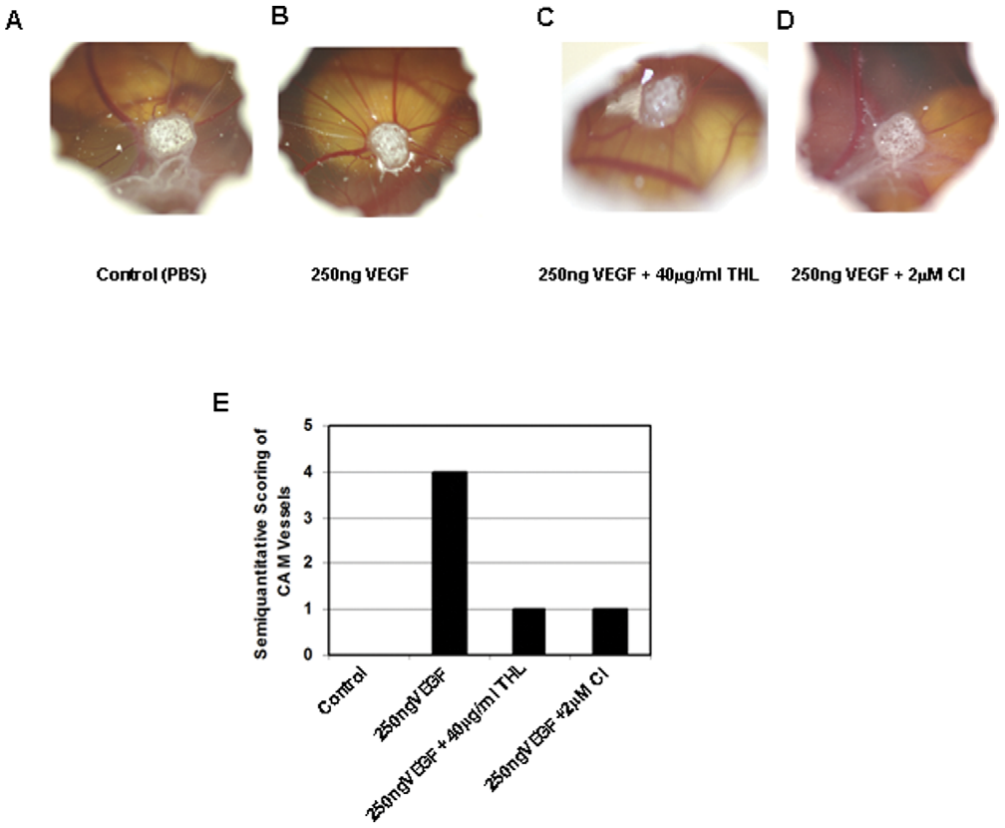
The effects of Triphala churna (THL) and chebulinic acid (CI) on vascular endothelial growth factor (VEGF) induced angiogenesis in the CAM assay. (**A, E**) PBS used as a control does not induce blood vessel formation. (**B, E**) VEGF induces new blood vessel formation. (**C, E**) THL inhibits VEGF induced new blood vessel formation (**D, E**) CI inhibits VEGF mediated new blood vessel formation. Representative photographs of six separate experiments are shown.

Similarly, to determine the quantity of CI in THL, 10 mg of THL powder was dissolved in 10 ml of distilled H_2_O and vigorously vortexed for 30 sec. The solution was then centrifuged at 2000 g for 3 min. Thereafter, the supernatant was transferred to a new vial and was diluted 1000X with mobile phase and finally, analyzed using the above mentioned LC-MS/MS method [6].

### Cell culture

Human umbilical vein endothelial cells (HUVEC) purchased from Lonza, CA, USA were maintained in EGM media supplemented with various growth factors and 2% FCS (Lonza, CA, USA). For *in vitro* experiments, HUVEC were serum and growth-factor starved for 24 h and thereafter, the effects of THL and CI were assessed [4], [18].

### *In vitro* toxicity assay

Trypan blue dye exclusion as a measure of cell viability was used to assess cytotoxicity of THL, and CI. Briefly, trypan blue (200 μl of 0.4% w/v dye) (Sigma, MO) was added to 2×10^4^ HUVEC and then the stained cells were counted at different time intervals after addition of different concentrations of the test compounds [19].

### Endothelial proliferation assay

HUVEC were seeded at a density of 5×10^3^ cells per well in 96 plates containing EGM media supplemented with various growth factors and 2% FCS till the cells were 70% confluent. These cells were serum and growth factor starved and were treated either with VEGF (20 ng/ml) or VEGF (20 ng/ml) + THL (40 μg/ml) or VEGF (20 ng/ml) + CI (2 μM) and incubated at 37°C for 24 hours. Thereafter proliferation of these cells was measured using Prestoblue™ Cell Viability reagent (Invitrogen, NY, USA) according to the manufacturer's protocol. Data values were measured as OD readings at 570/600 nm after addition and incubation with the reagent. Normalized 570 nm absorbance was calculated according to protocol of the manufacturer [20].

### Endothelial migration assay

*In vitro* wound healing assay was undertaken to evaluate the effects of THL and CI on VEGF induced HUVEC migration. The HUVEC were cultured to near confluence in 24-well plates containing EGM media supplemented with various growth factors and 2% FCS. Cells were serum and growth factors starved for 24 hours and then cell monolayers were wounded by a 200 μl pipette tip in one direction to create a scratch. The wounded cells were washed with PBS to remove cellular debris. To assess the effects of THL and CI on VEGF induced migration, cells were treated either with VEGF (20 ng/ml) or VEGF (20 ng/ml) + THL (40 μg/ml) or VEGF (20 ng/ml) + CI (2 μM) and incubated at 37°C for 18 hours. HUVEC migration was continuously monitored every two hours under a phase-contrast microscope after initial wounding till at 18 hours when no evident wound was observed in VEGF treated plates and wound closure was calculated as the distance covered by cells in relation to initial distance between two fronts and expressed as a percentage [18], [19].

### *In vitro* tube formation assay

*In vitro* tube formation was assessed using in vitro angiogenesis assay kit from Millipore, CA, USA as per the instructions of the manufacturer. Briefly, serum or growth factor starved HUVEC treated either with VEGF (20 ng/ml) or VEGF (20 ng/ml) + THL (40 μg/ml) or VEGF (20 ng/ml) + CI (2 μM) were seeded on extra cellular matrix and allowed to form capillary tube. The capillaries formed were observed on day 3 from the time of seeding using Carl Zeiss microscope [21].

### *In vitro* permeability assay

The assay was performed by using in vitro vascular permeability assay kit (Millipore, CA, USA) as per the manufacturer's protocol. Briefly, 2×10^5^ HUVEC in 200 μL were seeded onto collagen coated inserts in 24 well plates. 700 μL complete EBM medium was added to the plates. The cells were allowed to grow for 4–5 days until a monolayer was formed. The medium was replaced overnight with phenol free starving EBM medium. The respective inserts containing HUVEC were then pre-treated with 40 μg/ml of THL or 2 μM CI for 24 hrs. Subsequently, FITC-Dextran containing medium (phenol free EBM) with VEGF at 20 ng/ml was added. Fluorescence was measured after 60 min on the Spectra Flour Plus using excitation and emission wavelengths of 485 nm/535 nm [18], [22].

### Western Blot Analysis

This was performed using rabbit monoclonal antibody against phospho VEGFR-2 (Cell Signaling technology, MA, USA). Antibody-reactive bands were then detected by enzyme-linked chemiluminescence (Pierce Biotechnology, Inc.) and quantified by laser densitometry [17], [23].

### Chick chorioallantoic membrane (CAM) assay

The effects of THL and CI on VEGF induced angiogenesis was determined by semi-quantitative CAM assay. In order to expose CAM, a window was created in the shells of 3- day old Leghorn chicken eggs (OSU, Columbus, USA). These windows were sealed with a transparent tape. On day 8, 1-mm^3^ sterilized gelatin sponge (Pfizer, MI, USA) was asceptically inserted onto the CAM containing either PBS (control), or VEGF (250 ng) or VEGF (250 ng) + THL (40 μg/ml) or VEGF (250 ng) + CI (2 μM). Thereafter, angiogenesis was scored on day 12 as 0, negative; 0.5, change in vessel architecture; 1, partial spoke wheel (1/3 of circumference exhibits directional angiogenesis); 2, spoke wheel; 3 or greater, strong and fully spoke wheel [19], [24], [25]. Photographs were taken by Nikon D70 camera with AF Micro Nikkor 105 mm lens.

### Statistical analysis

All data are expressed as mean ± SEM. Differences among groups were evaluated by ANOVA and the unpaired Student's t test or Dunn's multiple comparison tests. P<0.05 was considered significant [4], [17].

## Results and Discussion

There are now studies which indicate the therapeutic efficacies of THL in tumor bearing animals [7]–[10]. However, there is still no report indicating the effects of THL on VEGF induced angiogenesis [7]–[10]. We at first determined whether single oral dose of 100 mg/kg of THL could inhibit VEGF (250 ng) mediated angiogenesis *in vivo* in a well established mouse matrigel plug assay model [14]–[17]. This dose of THL was particularly selected as this dose demonstrated the highest efficacy in human malignant tumor bearing mice [10]. In addition, we also did not observe any significant changes in the complete blood count, hepatic enzymes, cholesterol, blood sugar, blood urea nitrogen (BUN) and serum creatinine level with this dose of THL in mice when compared to normal controls (data not shown). On day 8, THL untreated plugs containing VEGF appeared dark red, Masson's trichrome staining (endothelial cells stain red and the matrigel stain blue) and CD31 immunostaining demonstrated higher levels of endothelial cells in these VEGF containing THL untreated plugs (Fig. 2 A–D). In contrast, on Day 8, plugs containing VEGF removed from animals treated with THL for 7 days were pale in color and the endothelial cells were also significantly less in numbers (Fig. 2A–D). Similar results were observed in control plugs without VEGF removed from animals untreated with THL (Fig. 2A–D). These data confirmed that oral administration of THL could significantly inhibit VEGF induced angiogenesis *in vivo*.

Furthermore *in vitro* studies have indicated the anti-VEGF actions of GA and EA, two constituents of THL [26], [27]. Since the bioavailability of these two compounds following ingestion of either fruits containing these two acids or in pure forms is poor [11], [12], [28], [29], [30] and because we had observed significant suppression of VEGF induced angiogenesis following oral administration of THL in our *in vivo* model (Fig. 2), we therefore examined the plasma level of another major constituent of THL, CI following oral feeding of mice with THL. The plasma concentration of CI reached to 1952.67 ng/ml (2.04 μM) at 20 min after gavaging the mice with a single dose of THL (100 mg/kg) containing 6.8 mg of CI as detected by LC-MS/MS.

Because VEGF mediates its angiogenic actions by stimulating proliferation, migration, tube formation and endothelial cell permeability [1]–[4], therefore in order to investigate whether THL could specifically inhibit these functions of VEGF in endothelial cells, we initially determined the non-toxic concentration of THL to be used for our *in vitro* experiments in HUVEC by examining the cytotoxic effects of various concentrations of THL (20–80 μg/ml) that were previously reported to inhibit tumor cell growth *in vitro* [9], [10], [31]. In addition, we also determined the effect of 2 μM of CI on the viability of HUVEC as this concentration of CI was detected in the plasma of mice after orally feeding them with the VEGF inhibitory dose of THL (100 mg/kg). Our results indicated 40 μg/ml of THL to be the highest non-toxic concentration of THL and 2 μM CI had no effect on cell viability (Fig. 3A, B). Accordingly, we selected 40 μg/ml of THL and 2 μM of CI for further *in vitro* experiments.

We next examined the effects of non-toxic concentration of THL (40 μg/ml) and CI (2 μM) on VEGF induced proliferation, migration, tube formation and permeability in HUVEC. Our results indicated significant inhibition of VEGF (20 ng/ml) induced proliferation (Fig. 3 C, D), migration (Fig. 4 A–D) and tube formation (Fig. 5A–D) by these cells after treatment with THL or CI. In addition, THL and CI also significantly inhibited VEGF induced permeability in HUVEC (Fig. 5E). It is to be noted here that THL (40 μg/ml) or CI (2 μM) alone had no effects on proliferation, wound healing, tube formation and permeability of the endothelial cells (data not shown).

Furthermore as these actions of VEGF is mediated mainly through its VEGFR-2 [1]–[4], therefore to elucidate the molecular mechanisms by which THL or CI inhibited VEGF functions, we investigated the effects of THL (40 μg/ml) and CI (2 μM) on VEGF (20 ng/ml) induced VEGFR-2 phosphorylation in HUVEC. Our results demonstrated that THL or CI significantly inhibited VEGF induced phosphorylation of VEGFR-2 (Fig. 5F).

Since our previous *in vitro* data suggested that THL and CI could significantly inhibit the important steps of VEGF induced angiogenesis (Fig. 3, 4, 5), therefore, we determined the effects of THL (40 μg/ml) and CI (2 μM) on VEGF mediated angiogenesis in CAM assay [19], [24], [25]. All observations were made on Day 4 after addition of these compounds. There was no evidence of angiogenesis or inflammation on addition of the vehicle (PBS) in which THL or CI were dissolved (Fig. 6A, E). However, striking angiogenesis was evident after exposure to 250 ng of VEGF (Fig. 6B, E). On the contrary, significant inhibition of VEGF induced angiogenesis was observed following exposures to 40 μg/ml of THL or 2 μM of CI (Fig. 6C, D, E). THL or CI alone did not induce any inflammation nor had any effects on blood vessel formation (data not shown).

Taken together our results for the first time demonstrated that THL or CI present in THL can significantly inhibit VEGF induced angiogenesis via suppression of VEGFR-2 actions. Moreover unlike the other constituents of THL such as GA and EA, the plasma level of CI reached considerably after oral intake of THL and this level of CI in turn could significantly and specifically inhibit the actions of VEGF *in vitro*. These results thus suggest that CI present in THL mediate the anti-VEGF effects of THL *in vivo* and is also a potent inhibitor VEGF functions. However, there may be other untested constituents of THL, which may also possess anti-VEGF activities.

Finally, VEGF mediated neovascularization plays an important pathogenic role in various diseases [1]–[3]. The presently available anti-VEGF drugs not only have serious toxicities, but are also very expensive [32]–[34]. This necessitates development of newer and effective non-toxic and inexpensive anti-VEGF agents. Our present study suggests that THL or CI may fulfill this promise in future.
